# All-paths graph kernel for protein-protein interaction extraction with evaluation of cross-corpus learning

**DOI:** 10.1186/1471-2105-9-S11-S2

**Published:** 2008-11-19

**Authors:** Antti Airola, Sampo Pyysalo, Jari Björne, Tapio Pahikkala, Filip Ginter, Tapio Salakoski

**Affiliations:** 1 Turku Centre for Computer Science (TUCS) and the Department of IT, University of Turku, Joukahaisenkatu 3-5, 20520 Turku, Finland

## Abstract

**Background:**

Automated extraction of protein-protein interactions (PPI) is an important and widely studied task in biomedical text mining. We propose a graph kernel based approach for this task. In contrast to earlier approaches to PPI extraction, the introduced all-paths graph kernel has the capability to make use of full, general dependency graphs representing the sentence structure.

**Results:**

We evaluate the proposed method on five publicly available PPI corpora, providing the most comprehensive evaluation done for a machine learning based PPI-extraction system. We additionally perform a detailed evaluation of the effects of training and testing on different resources, providing insight into the challenges involved in applying a system beyond the data it was trained on. Our method is shown to achieve state-of-the-art performance with respect to comparable evaluations, with 56.4 F-score and 84.8 AUC on the AImed corpus.

**Conclusion:**

We show that the graph kernel approach performs on state-of-the-art level in PPI extraction, and note the possible extension to the task of extracting complex interactions. Cross-corpus results provide further insight into how the learning generalizes beyond individual corpora. Further, we identify several pitfalls that can make evaluations of PPI-extraction systems incomparable, or even invalid. These include incorrect cross-validation strategies and problems related to comparing F-score results achieved on different evaluation resources. Recommendations for avoiding these pitfalls are provided.

## Background

Information extraction from biomedical research publications has been a topic of intense research during recent years [1-3]. Literature databases such as PubMed offer access through online interfaces to records of millions of research articles from the biomedical domain, with abstracts made available for many, and full texts for some of the papers. Potentially, this offers a researcher direct access to vast amounts of research knowledge. However, locating the useful information can be challenging, a simple keyword search may still return many more articles than a human being can process. This motivates the development of tools for automating the extraction of information from biomedical text.

A task of significant interest in biomedical natural language processing is the automated protein-protein interaction (PPI) extraction. The most commonly addressed problem has been the extraction of binary interactions, where the system identifies which protein pairs in a sentence have a biologically relevant relationship between them. Proposed solutions include both hand-crafted rule-based systems and machine learning approaches (see e.g. [4]). A wide range of results have been reported for the systems, but as we will show, differences in evaluation resources, metrics and strategies make direct comparison of the numbers presented problematic. Further, the results gained from the BioCreative II evaluation, where the best performing system achieved a 29% F-score [5], suggest that the problem of extracting binary protein-protein interactions is far from solved.

The public availability of large annotated PPI-corpora such as AImed [4], BioInfer [6] and GENIA [7], provides an opportunity for building PPI extraction systems automatically using machine learning. A major challenge is how to supply the learner with the contextual and syntactic information needed to distinguish between interactions and non-interactions. To address the ambiguity and variability of the natural language expressions used to state PPI, several recent studies have focused on the development, adaptation and application of NLP tools for the biomedical domain. Many high-quality domain-specific tools are now freely available, including full parsers such as that introduced by Lease and Charniak [8]. Additionally, a number of conversions from phrase structure parses to dependency structures that make the relationships between words more directly accessible have been introduced. These include conversions into representations such as the Stanford dependency scheme [9] that are explicitly designed for information extraction purposes. However, specialized feature representations and kernels are required to make learning from such structures possible.

**Figure 1:**
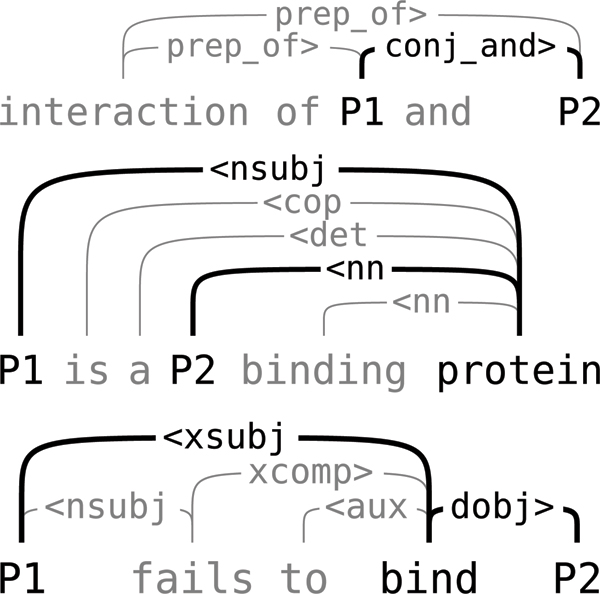
**Shortest path example**. Stanford dependency parses ("collapsed") representation where the shortest path, shown in bold, excludes important words.

Approaches such as subsequence kernels [10], tree kernels [11] and shortest path kernels [12] have been proposed and successfully used for relation extraction. However, these methods lack the expressive power to consider representations derived from general, possibly cyclic, dependency graph structures, such as those generated by the Stanford tools. The subsequence kernel approach does not consider parses at all, and the shortest path approach is limited to representing only a single path in the full dependency graph, which excludes relevant words even in many simple cases (Figure 1). Tree kernels can represent more complex structures, but are still restricted to tree representations.

Lately, in the framework of kernel-based machine learning methods there has been an increased interest in designing kernel functions for graph data. Building on the work of Gärtner et al. [13], graph representations tailored for the task of dependency parse ranking were proposed by Pahikkala et al. [14]. Though the proposed representations are not directly applicable to the task of PPI extraction, they offer insight in how to learn from dependency graphs. We develop a graph kernel approach for PPI extraction based on these ideas.

We next define a graph representation suitable for describing potential interactions and introduce a kernel which makes efficient learning from a general, unrestricted graph representation possible. Then we provide a short description of the sparse regularized least squares (sparse RLS) kernel-based machine learning method we use for PPI-extraction.

Further, we rigorously assess our method on five publicly available PPI corpora. In addition to purely intrinsic evaluation using cross-validation on single corpora, we provide a broad cross-corpus evaluation to test how well an extraction system trained on a given corpus will generalize to the other corpora. We thus provide, to our knowledge, the most comprehensive evaluation done with a machine learning approach to PPI-extraction. Finally, we discuss the effects that different evaluation strategies, choice of corpus and applied metrics have on measured performance, and provide conclusions.

## Methods

We next present our graph representation, formalize the notion of graph kernels, and present our learning method of choice, the sparse RLS.

### Graph encoding of sentence structure

As in most recent work on machine learning for PPI extraction, we cast the task as learning a decision function that determines for each unordered candidate pair of protein names occurring together in a sentence whether the two proteins interact. In the following, we first define the graph representation used to represent an interaction candidate pair. We then proceed to derive the kernel used to measure the similarities of these graphs.

**Figure 2:**
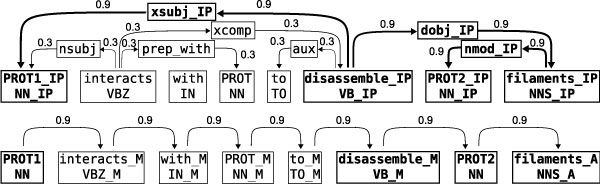
**Graph representation**. Graph representation generated from an example sentence. The candidate interaction pair is marked as PROT1 and PROT2, the third protein is marked as PROT. The shortest path between the proteins is shown in bold. In the dependency based subgraph all nodes in a shortest path are specialized using a post-tag (IP). In the linear order subgraph possible tags are (B)efore, (M)iddle, and (A)fter. For the other two candidate pairs in the sentence, graphs with the same structure but different weights and labels would be generated.

We assume that the input of our learning method is a dependency parse of a sentence where a pair of protein names is marked as the candidate interaction for which an extraction decision must be made. Based on this, we form a weighted, directed graph that consists of two unconnected subgraphs. One represents the dependency structure of the sentence, and the other the linear order of the words (see Figure 2).

The first subgraph is built from the dependency analysis. One vertex and an associated set of labels is created in the graph for each token and for each dependency. The vertices that represent tokens have as labels the text and part-of-speech (POS) of the token. To ensure generalization of the learned extraction model, the labels of vertices that correspond to protein names are replaced with PROT1, PROT2 or PROT, where PROT1 and PROT2 are the pair of interest. The vertices that represent dependencies are labeled with the type of the dependency. The edges in the subgraph are defined so that each dependency vertex is connected by an incoming edge from the vertex representing its governor token, and by an outgoing edge to the vertex representing its dependent token. The graph thus represents the entire sentence structure.

It is widely acknowledged that the words between the candidate entities or connecting them in a syntactic representation are particularly likely to carry information regarding their relationship; Bunescu and Mooney [12] formalize this intuition for dependency graphs as the *shortest path hypothesis*. We apply this insight in two ways in the graph representation: the labels of the nodes on the shortest undirected paths connecting PROT1 and PROT2 are differentiated from the labels outside the paths using a special tag. Further, the edges are assigned weights; after limited preliminary experiments, we chose a simple weighting scheme where all edges on the shortest paths receive a weight of 0.9 and other edges receive a weight of 0.3. The representation thus allows us to emphasize the shortest path without completely disregarding potentially relevant words outside the path.

The second subgraph is built from the linear structure of the sentence. For each token, a second vertex is created and the labels for the vertices are derived from the texts, POS-tags and named entity tagging as above. The labels of each word are specialized to denote whether the word appears before, in-between, or after the protein pair of interest. Each word node is connected by an edge to its succeeding word, as determined by sentence order of the words. Each edge is given the weight 0.9.

### The all-paths graph kernel

We next formalize the graph representation and present the all-paths graph kernel. This kernel can be considered as a practical instantiation of the theoretical graph kernel framework introduced by Gärtner et al. [13]. Let *V *be the set of vertices in the graph and ℒ be the set of possible labels vertices can have. We represent the graph with an adjacency matrix *A *∈ ℝ^|*V*| × |*V*|^, whose rows and columns are indexed by the vertices, and [*A*]_*i*, *j *_contains the weight of the edge connecting *v*_*i *_∈ *V *and *v*_*j *_∈ *V *if such an edge exists, and zero otherwise. Further, we represent the labels as a label allocation matrix $L\in {\mathbb{R}}^{\left|ℒ|\times |V\right|}$ so that *L*_*i*, *j *_= 1 if the *j*-th vertex has the *i*-th label and *L*_*i*, *j *_= 0 otherwise. Because only a very small fraction of all the possible labels are ever assigned to any single node, this matrix is extremely sparse.

It is well known that when an adjacency matrix is multiplied with itself, each element [*A*^2^]_*i*, *j *_contains the summed weight of paths from vertex *v*_*i *_to vertex *v*_*j *_through one intervening vertex, that is, paths of length two. Similarly, for any length *n*, the summed weights from *v*_*i *_to *v*_*j *_can be determined by calculating [*A*^*n*^]_*i*, *j*_. Since we are interested not only in paths of one specific length, it is natural to combine the effect of paths of different lengths by summing the powers of the adjacency matrices. We calculate the infinite sum of the weights of all possible paths connecting the vertices using the Neumann Series, defined as

$${\left(I-A\right)}^{-1}=I+A+A^{2}+\ldots =\sum_{k=0}^{\infty }A^{k}$$

if |*A*| < 1 where |*A*| is the spectral radius of *A *[15]. From this sum we can form a new adjacency matrix

*W *= (*I *- *A*)^-1 ^- *I*.

The final adjacency matrix contains the summed weights of all possible paths connecting the vertices. The identity matrix is subtracted to remove the paths of length zero, which would correspond to self-loops. Next, we present the graph kernel that utilizes the graph representation defined previously. We define an instance *G *representing a candidate interaction as *G *= *LWL*^T^, where *L *and *W *are the label allocation matrix and the final adjacency matrix corresponding to the graph representation of the candidate interaction.

Following Gärtner et al. [13] the graph kernel is defined as

$$k(G^{\prime },G^{\prime \prime })=\sum_{i=1}^{\left|ℒ\right|}\sum_{j=1}^{\left|ℒ\right|}{G^{\prime }}_{i,j}{G^{\prime \prime }}_{i,j},$$

where *G' *and *G" *are two instances formed as defined previously. The features can be thought as combinations of labels from connected pairs of vertices, with a value that represents the strength of their connection. In practical implementations, the full *G *matrices, which consist mostly of zeroes, are never explicitly formed. Rather, only the non-zero elements are stored in memory and used when calculating the kernels.

### Scalable learning with Sparse RLS

RLS, also known as the least squares support vector machine, is a state-of-the-art kernel-based machine learning method which has been shown to have comparable performance to standard support vector machines [16,17]. We choose the sparse version of the algorithm, also known as subset of regressors, as it allows us to scale up the method to very large training set sizes. Sparse RLS also has the property that it is possible to perform cross-validation and regularization parameter selection so that their time complexities are negligible compared to the training complexity. These efficient methods are analogous to the ones proposed by Pahikkala et al. [18] for the basic RLS regression.

We now briefly present the basic sparse RLS algorithm. Let *m *denote the training set size and *M *= {1,..., *m*} an index set in which the indices refer to the examples in the training set. Instead of allowing functions that can be expressed as a linear combination over the whole training set, as in the case of basic RLS regression, we only allow functions of the following restricted type:

(1) $$f\left(\cdot \right)=\sum_{i\in B}a_{i}k\left(\cdot ,x_{i}\right),$$

where *k *is the kernel function, *x*_*i *_are training data points, *a*_*i *_∈ ℝ are weights, and the set indexing the basis vectors *B *⊂ *M *is selected in advance. The coefficients *a*_*i *_that determine (1) are obtained by minimizing

(2) $$\sum_{j=1}^{m}(y_{j}-\sum_{i\in B}a_{i}k(x_{j},x_{i}){)}^{2}+\lambda \sum_{j,i\in B}a_{j}a_{i}k(x_{j},x_{i}),$$

where the first term is the squared loss function, the second term is the regularizer, and *λ *∈ ℝ_+ _is a regularization parameter. All the training instances are used for determining the coefficient vector, but only a subset of them to represent the learned hypothesis. The minimizer of (2) is obtained by solving the corresponding system of linear equations, which can be performed in *O*(*m*|*B*|^2^) time.

We set the maximum number of basis vectors to 4000 in all experiments in this study. The subset is selected randomly when the training set size exceeds this number. Other methods for the selection of the basis vectors were considered by Rifkin et al. [17], who however reported that the random selection worked as well as the more sophisticated approaches.

## Results and discussion

We next describe the evaluation resources and metrics used, provide a comprehensive evaluation of our method across five PPI corpora, and compare our results to earlier work. Further, we discuss the challenges inherent in providing a valid method evaluation and propose solutions.

### Corpora and evaluation criteria

We evaluate our method using five publicly available corpora that contain PPI interaction annotation: AImed [4], BioInfer [6], HPRD50 [19], IEPA [20] and LLL [21]. All the corpora were processed to a common format using transformations [22] that we have introduced earlier [23]. We note that the version of the BioInfer used in this study differs from the one we considered in [23] and in [24]. This is due to the fact that these studies used an early version of the binarization rules [25] that transform the complex relations of BioInfer to binary ones.

We parse these corpora with the Charniak-Lease parser [8], which has been found to perform best among a number of parsers tested in recent domain evaluations [26,27]. The Charniak-Lease phrase structure parses are transformed into the collapsed Stanford dependency scheme using the Stanford tools [9]. We cast the PPI extraction task as binary classification, where protein pairs that are stated to interact are positive examples and other co-occurring pairs negative. Thus, from each sentence, $\left(\begin{array}{c} n\\ 2 \end{array}\right)$ examples are generated, where *n *is the number of occurrences of protein names in the sentence. Finally, we form the graph representation described earlier for each candidate interaction.

In the single corpus tests we evaluate the method with 10-fold document-level cross-validation on all of the corpora. This guarantees the maximal use of the available data, and also allows comparison to relevant earlier work. In particular, on the AImed corpus we apply the exact same 10-fold split that was used by Bunescu et al. [10], Giuliano et al. [28], Van Landeghem et al. [29], and possibly some of the other studies which do not explicitly state which split was used. In cross-corpus tests we use each of the corpora in turn to train an extraction system, and test the system on the four remaining corpora.

Performance is measured according to the following criteria: interactions are considered untyped, undirected pairwise relations between specific protein mentions, that is, if the same protein name occurs multiple times in a sentence, the correct interactions must be extracted for each occurrence. Further, we do not consider self-interactions as candidates and remove them from the corpora prior to evaluation. The majority of PPI extraction system evaluations use the balanced F-score measure for quantifying the performance of the systems. This metric is defined as $F=\frac{2pr}{p+r}$, where *p *is precision and *r *recall. Likewise, we provide F-score, precision, and recall values in our evaluation. It should be noted that F-score is very sensitive to the underlying positive/negative pair distribution of the corpus – a property whose impact on evaluation is discussed in detail below. As an alternative to F-score, we also evaluate the performance of our system using the *area under the receiver operating characteristics curve *(AUC) measure [30]. AUC has the important property that it is invariant to the class distribution of the used dataset. Due to this and other beneficial properties for comparative evaluation, the usage of AUC for performance evaluation has been recently advocated in the machine learning community (see e.g. [31]). Formally, AUC can be defined as

$$AUC=\frac{\sum_{i=1}^{m_{+}}\sum_{j=1}^{m_{-}}H(x_{i}-y_{j})}{m_{+}m_{-}},$$

where *m*_+ _and *m*_- _are the numbers of positive and negative examples, respectively, and *x*_1_,...,$x_{m_{+}}$ are outputs of the system for the positive, and *y*_1_,...,$y_{m_{-}}$ for the negative examples, and

$$H(r)=\{\begin{array}{ll} 1, & \text{if }r>0\\ 0.5, & \text{if }r=0\\ 0, & \text{otherwise}. \end{array}$$

The outputs are real valued and can be thought of as inducing a ranking, where the examples considered to be most likely to belong to the positive class should receive the highest output values. The measure corresponds to the probability that given a randomly chosen positive and negative example, the system will be able to correctly distinguish which one is which.

### Performance on the individual corpora

The performance of our method on the five corpora for the various metrics is presented in Table 1. For reference, we show also the performance of the co-occurrence (or *all-true*) baseline, which simply assigns each candidate into the interaction class. The recall of the co-occurrence method is trivially 100%, and in terms of AUC it has a performance of 50%, the random baseline. All the numbers in Table 1, including the co-occurrence results, are averages taken over the ten folds. One should note that because of the non-linearity of the F-score measure, the average precision and recall will not produce exactly the average F. Further, calculating the co-occurrence numbers as averages over the folds leads to results that differ slightly compared to the approach where the co-occurrence statistic is calculated over all the data pooled together.

**Table 1:** Evaluation results. Counts of positive and negative examples in the corpora and (P)recision, (R)ecall, (F)-score and AUC for the graph kernel, with standard deviations provided for F and AUC.

	Statistics			Graph Kernel							co-occ	
Corpus	#POS.	#NEG.		P	R	F	*σ*_*F*_	AUC	*σ*_*AUC*_		P	F
AIMed	1000	4834		52.9%	61.8%	56.4%	5.0%	84.8%	2.3%		17.8%	30.1%
BioInfer	2534	7132		56.7%	67.2%	61.3%	5.2%	81.9%	4.9%		26.6%	41.7%
HPRD50	163	270		64.3%	65.8%	63.4%	11.4%	79.7%	6.3%		38.9%	55.4%
IEPA	335	482		69.6%	82.7%	75.1%	7.0%	85.1%	5.1%		40.8%	57.6%
LLL	164	166		72.5%	87.2%	76.8%	17.8%	83.4%	12.2%		55.9%	70.3%

The results hold several interesting findings. First, we briefly observe that on the AImed corpus, which has recently been applied in numerous evaluations [32] and can be seen as an emerging *de facto *standard for PPI extraction method evaluation, the method achieves an F-score performance of 56.4%. As we argue in more detail below, this level of performance is comparable to the state-of-the-art in machine learning based PPI extraction. For the other large corpus, BioInfer, F-score performance is somewhat higher, at 61%. Second, we observe that the F-score performance of the method varies strikingly between the different corpora, with results on IEPA and LLL approximately 20 percentage units higher than on AImed and 15 percentage units higher than on BioInfer, despite the larger size of the latter two. In our previous work we have observed similar results with a rule-based extraction method [23]. As a broad multiple corpus evaluation using a state-of-the-art machine learning method for PPI extraction, our results support and extend the key finding that F-score performance results measured on different corpora cannot, in general, be meaningfully compared.

The co-occurrence baseline numbers indicate one reason for the high F-score variance between the corpora. The F-score metric is not invariant to the distribution of positive and negative examples: for example, halving the number of negative test examples is expected to approximately halve the number of false positives at a given recall point. Thus, the greater the fraction of true interactions in a corpus is, the easier it is to reach high performance in terms of F-score. This is reflected in co-occurrence results, which range from 30% to 70% depending on the class distribution of the corpus.

This is a critical weakness of the F-score metric in comparisons involving different corpora as, for example, the fraction of true interactions out of all candidates is 50% in the LLL corpus but only 17% in AImed. By contrast to the large differences in performance measured using F-score, we find that for the distribution-invariant AUC measure the performance for all of the corpora falls in the range of 80–85%. The results provide an argument in favor of applying the AUC metric instead of, or in addition to, F-score. AUC is also more stable in terms of variance.

**Figure 3:**
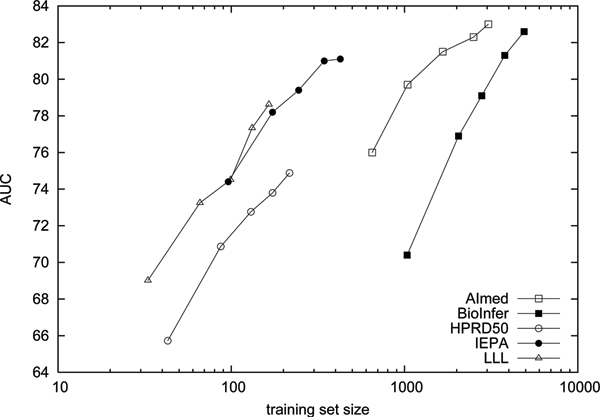
**Learning curves**. Learning curves for the five corpora. The scale is logarithmic with respect to the amount of training data.

The similar performance in terms of AUC for corpora with as widely differing sizes as LLL and BioInfer allows for two alternative interpretations. First, it might be that past a relatively modest number of examples, increasing corpus size has little effect on the performance of the method. Alternatively, it might be the case that the larger corpora, while having more training data available, are also more difficult to learn than the smaller corpora. We explore the issue further by calculating learning curves on the corpora, using AUC as the performance measure (see Figure 3). For each corpus five folds are set aside as the test set, and the rest of the data is incrementally added to the training set to test how increase in training data affects the performance.

The learning curves support the latter interpretation. If the datasets all represented equally difficult tasks with respect to distinguishing randomly drawn positive instances from negatives, we would expect the curves to roughly overlap. The fact that they are to a large extent separate indicates that there are large differences in the difficulty of the learning tasks represented by the different corpora. On AImed and BioInfer it takes significantly more data to reach the same performance than on the three smaller corpora HPRD50, LLL and IEPA: For example, performance on the latter three with 100 training examples exceeds the performance on BioInfer with ten times as much training data.

### Cross-corpus performance

The cross-corpus evaluation aims to shed light on a question of fundamental importance in training machine learning based PPI-extraction systems: Will the learned models generalize beyond the specific characteristics of the data they were trained on? The types of named entities annotated, the definition of what exactly constitutes an interaction and the relative positive/negative distributions of pairs can vary significantly over different corpora. Thus it is not obvious that a system trained on a given corpus will perform well on data which is not from the same corpus. As discussed in [33], applying text mining tools beyond the development data can lead to disappointing results.

We explore this issue through a cross-corpus evaluation of our method. Five extraction systems are trained, one on each corpus, and they are each tested on the four remaining corpora. Leave-one-document-out cross-validation on the training corpus is used for parameter value selection. Our evaluation extends the recent results of Van Landeghem et al. [29], who conducted cross-corpus experiments on four of the corpora considered in this study. Their finding was that models trained on a combination of three of the corpora often did not perform well on terms of F-score, when tested on the remaining corpus.

We start by considering the AUC results of the cross-corpus evaluation (see Table 2), as the metric normalizes away much of the differences resulting from differing positive/negative distributions and threshold selection strategies, thus providing a more stable view of performance. We notice that the performance varies significantly depending on the training and test corpus. Unlike in the single corpus evaluations the results are scattered, ranging from 61% to 83% AUC. On the large corpora the trained extraction systems in all cases perform clearly worse than the cross-validation performance. However, on the two smallest corpora this is not so. On HPRD50 systems trained on AImed and IEPA actually give better performance than the results from cross-validating on the corpus. On LLL the models trained on BioInfer and IEPA do almost as well as the cross-validation results on the corpus. These results suggest that a larger amount of training data can compensate for the differences in corpus annotation strategies to a large extent. Random chance may also be a factor here, as observed previously in the large variances in cross-validation results on the smallest corpora.

**Table 2:** Cross-corpus results measured with AUC. AUC results for cross-corpus testing. Rows correspond to training corpora and columns to test corpora.

	AImed	rank	BioInfer	rank	HPRD50	rank	IEPA	rank	LLL	rank	avg. rank
AImed	-	-	67.7%	2	82.4%	1	76.1%	2	77.8%	3	2
BioInfer	77.8%	1	-	-	75.2%	3	79.3%	1	83.3%	1	1.5
HPRD50	72.5%	2	61.8%	3	-	-	74.9%	3	64.0%	4	3
IEPA	70.2%	3	72.2%	1	80.0%	2	-	-	82.5%	2	2
LLL	61.8%	4	61.0%	4	69.4%	4	74.8%	4	-	-	4

One relevant question that can be answered from the cross-corpus experiments is which of the corpora provides the best resource for training from a generalization perspective. However, it is not entirely straightforward to meaningfully summarize these results: simple averages over results on the very different resources carry little meaning. Instead, we provide a simple, rough indicator of generalization potential by ranking the corpora separately according to the results on each of the other corpora. The rankings are presented in Table 2. Though the rankings do differ over different test corpora, overall they roughly follow the size of the corpora. On average models trained on the largest corpus, BioInfer, perform best. Next in this ranking the second and third largest corpora, AImed and IEPA share a rank. The second worst performing models are trained on the second smallest corpus HPRD50, and the lowest performing ones on the smallest dataset, LLL. Unsurprisingly, the more training data available the better the performance is. A surprising result is the high performance of systems trained on IEPA, the corpus being an order of magnitude smaller than AImed or BioInfer.

Next, we consider the results using the F-score measure. In Table 3 results for which the threshold separarating positive and negative classes has been selected on the training corpus are shown. In some cases the results are on a similar level to those gained in the single corpus cross-validation experiments. This holds true for example with models trained on AImed or IEPA, and tested on the HPRD50 corpus. However, there are several cases where the performance is disastrously low. Most strikingly, three out of four results gained when using AImed for training fall below the results one would achieve with the naive co-occurrence baseline. We observe that even in these cases the AUC results are still competitive. This gives rise to the assumption that the problem is in the threshold selection. The learned models do have the property that they tend to assign higher values for the positive than for the negative examples, but the approach of selecting the suitable threshold on training data for separating the two classes fails utterly in some cases. We further observed that avoiding the task of threshold selection altogether by setting it simply to zero yielded no better results.

**Table 3:** Cross-corpus results measured with F-score and threshold chosen on training set. F-score results for cross-corpus testing with the thresholds chosen on the training set. Rows correspond to training corpora and columns to test corpora. Δ denote the difference between the F-score result and the result achieved with the optimal threshold.

	AImed	Δ	BioInfer	Δ	HPRD50	Δ	IEPA	Δ	LLL	Δ
AImed	-	-	24.9%	22.2%	64.6%	4.4%	22.9%	44.5%	17.7%	56.8%
BioInfer	44.2%	3.0%	-	-	63.6%	0.3%	64.5%	3.5%	76.4%	1.6%
HPRD50	40.9%	1.3%	27.2%	15.3%	-	-	56.3%	8.8%	45.5%	22.4%
IEPA	38.4%	0.7%	47.0%	4.7%	65.6%	1.9%	-	-	77.0%	0.6%
LLL	32.6%	0.7%	42.2%	0.3%	58.3%	1.5%	63.9%	1.0%	-	-

In Table 4 we provide the optimal F-score results, choosing the positions from the precision/recall curves that would lead to highest F-scores. Many of the results are now greatly increased, with no result falling below the naive co-occurrence baseline. Further, the relative ranking order of the results is the same as that induced by the AUC scores. It is now clear that one can not necessarily rely on the approach of choosing the threshold according to what works on the training set when doing cross-corpus learning. This is perhaps due to the large differences in the underlying positive/negative distributions of the corpora. The differences mean breaking the basic assumption made by the majority of machine learning methods, that the training and test examples are identically distributed. As can be seen from the statistics presented in Table 1, the examples are clearly not identically distributed over the corpora, at least with respect to outputs.

**Table 4:** Cross-corpus results measures with F-score and optimal thresholds. F-score results for cross-corpus testing with the optimal thresholds. Rows correspond to training corpora and columns to test corpora.

	AImed	BioInfer	HPRD50	IEPA	LLL
AImed	-	47.1%	69.0%	67.4%	74.5%
BioInfer	47.2%	-	63.9%	68.0%	78.0%
HPRD50	42.2%	42.5%	-	65.1%	67.9%
IEPA	39.1%	51.7%	67.5%	-	77.6%
LLL	33.3%	42.5%	59.8%	64.9%	-

One approach for selecting which examples to assign to positive and which to negative classes could be selecting the threshold according to the the relative positive/negative distribution of the test set. To estimate this in a practical setting, one may have to sample and manually check examples from the test set. In Table 5 are presented the F-score results gained when assigning to positive class such a fraction of the test examples that corresponds to the relative frequency of positive examples in the test corpus. In all the cases the results are within a few percentage units of the optimal values, indicating that this simple heuristic allows the worst disasters observed in the cross-corpus tests to be avoided. However, there are several cases where the result achieved with this approach is lower than when choosing the threshold on the training data.

**Table 5:** Cross-corpus results measured with F-score and thresholds based on the distribution of test set. F-score results for cross-corpus testing with the thresholds chosen according to the positive/negative distribution of the test set. Rows correspond to training corpora and columns to test corpora. Δ denote the difference between the F-score result and the result achieved with the optimal threshold.

	AImed	Δ	BioInfer	Δ	HPRD50	Δ	IEPA	Δ	LLL	Δ
AImed	-	-	44.7%	2.4%	65.6%	3.4%	63.9%	3.5%	70.1%	4.4%
BioInfer	42.6%	4.6%	-	-	62.0%	1.9%	66.9%	1.1%	75.6%	2.4%
HPRD50	39.1%	3.1%	40.0%	2.5%	-	-	63.3%	1.8%	58.5%	9.4%
IEPA	33.5%	5.6%	48.4%	3.3%	66.3%	1.2%	-	-	77.4%	0.2%
LLL	26.5%	6.8%	38.7%	3.8%	54.0%	5.8%	63.0%	1.9%	-	-

To conclude, the cross-corpus learning results support the assumption that the learned models generalize beyond the corpora they were trained on. Still, results are generally lower when testing a method against a corpus different from that on which it was trained. We observe that the systems trained on larger corpora tend to perform better than the ones trained on smaller ones, as is to be expected. The results achieved with the IEPA as a training corpus are surprisingly competitive, considering how much smaller it is than the two larger corpora. Choosing a threshold for separating the positive and negative classes proves to be a challenging issue, as a threshold chosen on the training corpus may not work at all on another.

### Performance compared to other methods

We next discuss the performance of our method compared to other methods introduced in the literature and the challenges of meaningful comparison, where we identify three major issues.

First, as indicated by the results above, differences in the makeup of different corpora render cross-corpus comparisons in terms of F-score essentially meaningless. As F-score is typically the only metric for which results are reported in the PPI extraction literature, we are limited to comparing against results on single corpora. We consider the AImed and BioInfer evaluations to be the most relevant ones, as these corpora are sufficiently large for training and reliably testing machine learning methods. As the present study is, to the best of our knowledge, the first to report machine learning method performance on BioInfer, we will focus on AImed in the following comparison.

Second, the cross-validation strategy used in evaluation has a large impact on measured performance. The pair-based examples can break the assumption of the training and test sets being independent of each other, as pairs generated from the same sentence, and to a lesser extent from the same document, are clearly not independent. This must be taken into account when designing the experimental setup (see e.g. [18] for further discussion). In earlier system evaluations, two major strategies for defining the splits used in cross-validation can be observed. The approach used by Bunescu and Mooney [10], which we consider the correct one, is to split the data into folds on the level of documents. This guarantees that all pairs generated from the same document are always either in the training set or in the test set. Another approach is to pool all the generated pairs together, and then randomly split them to folds. To illustrate the significance of this choice, consider two interaction candidates extracted from the same sentence, e.g. from a statement of the form "*P*_1 _and *P*_2 _[...] *P*_3_", where "[...]" is any statement of interaction or non-interaction. Due to the near-identity of contexts, a machine learning method will easily learn to predict that the label of the pair (*P*_1_, *P*_3_) should match that of (*P*_2_, *P*_3_). However, such "learning" will clearly not generalize. This approach must thus be considered invalid, because allowing pairs generated from the same sentences to appear in different folds leads to an information leak between the training and test sets. Sætre et al. [32] observed that adopting the latter cross-validation strategy on AImed could lead *up to 18 F-score percentage unit overestimation of performance*. For this reason, we will not consider results listed in the "False 10-fold cross-validation" table (2b) of Sætre et al. [32].

With these restrictions in place, we now turn to comparison with relevant results reported in related research, summarized in Table 6. Among the work left out of the comparison we note the results of Bunescu and Mooney [10], who reported a performance of 54.2% F on AImed. Though they used the same cross-validation strategy as the one used in our experiments, their results are not comparable to the ones included in the Table 6. They applied evaluation criteria where it is enough to extract only one occurrence of each mention of an interaction from each abstract, while the results shown were evaluated using the same criteria as applied here. The former approach can produce higher performance: the evaluation of Giuliano et al. [28] includes both alternatives, and their method achieves an F-score of 63.9% under the former criterion, which they term One Answer per Relation in a given Document (OARD).

**Table 6:** Comparison on AImed. (P)recision, (R)ecall, (F)-score and AUC results for methods evaluated on AImed with the correct cross-validation methodology. Note that the best performing method, introduced by Miwa et al. [34], also utilizes the all-paths graph kernel.

	P	R	F	AUC
Miwa et al. [34]	-	-	63.5%	87.9%
Miyao et al. [35]	54.9%	65.5%	59.5%	-
Giuliano et al. [28]	60.9%	57.2%	59.0%	-
All-paths graph kernel	52.9%	61.8%	56.4%	84.8%
Sætre et al. [32]	64.3%	44.1%	52.0%	-
Mitsumori et al. [39]	54.2%	42.6%	47.7%	-
Van Landeghem et al. [29]	49%	44%	46%	-
Yakushiji et al. [40]	33.7%	33.1%	33.4%	-

The best performing system, that of Miwa et al. [34], combines the all-paths graph kernel, implemented based on the description we provided in [24], together with other kernels. Their results can be considered as a further validation about the suitability of the graph kernel for PPI-extraction. Our implementation of the all-paths method outperforms most of the other studies using similar evaluation methodology, with the exceptions being the approaches Miyao et al. [35] and Giuliano et al. [28].

Miyao reports choosing in the experiments always the optimal point from the precision/recall curve, an approach we observe would raise our results around the same level. The results of Giuliano et al. are somewhat surprising, as their method does not apply any form of parsing but relies instead only on the sequential order of the words. This brings us to our third point regarding comparability of methods. As pointed out by Sætre et al. [32], the AImed corpus allows remarkably different "interpretations" regarding the number of interacting and non-interacting pairs. For example, where we have identified 1000 interacting and 4834 non-interacting protein pairs in AImed, in the data used by Giuliano there are eight more interacting and 200 fewer non-interacting pairs. The corpus can also be preprocessed in a number of ways. In particular we noticed that whereas protein names are always blinded in our data, in the data used by Giuliano protein names are sometimes partly left visible. As Giuliano has generously made his method implementation available [36], we were able to test the performance of his system on the data we used in our experiments. This resulted in an F-score of 52.4%.

Finally, there remains an issue of parameter selection. For sparse RLS the values of the regularization parameter λ and the decision threshold separating the positive and negative classes must be chosen, which can be problematic when no separate data for choosing them is available. Choosing from several parameter values the ones that give best results in testing, or picking the best point from a precision/recall curve when evaluating in terms of F-score, will lead to an over-optimistic evaluation of performance. This issue has often not been addressed in earlier evaluations that do cross-validation on a whole corpus. We choose the parameters by doing further leave-one-document-out cross-validation within each round of 10-fold-cross-validation, on the nine folds that constitute the training set.

As a conclusion, we observe the results achieved with the all-paths graph kernel to be state-of-the-art level. However, differences in evaluation strategies and the large variance exhibited in the results make it impossible to state which of the systems considered can be expected in general to perform best. We encourage future PPI-system evaluations to report AUC and F-score results over multiple corpora, following clearly defined evaluation strategies, to bring further clarity to this issue. For further discussion on resolving the challenges of comparing biomedical relation extraction results we refer to [37].

## Conclusion

In this paper we have proposed a graph kernel approach to extracting protein-protein interactions, which captures the information in unrestricted dependency graphs to a format that kernel based learning algorithms can process. The method combines syntactic analysis with a representation of the linear order of the sentence, and considers all possible paths connecting any two vertices in the resulting graph. We demonstrate state-of-the-art performance for the approach. All software developed in the course of this study is made publicly available at [22].

A cross-corpus evaluation is performed to test whether an extraction system will work beyond the corpus it was trained on. We observe this to be the case, though results are generally worse than when training and testing on data from the same corpus. Having a larger amount of data available leads to better performance. Extraction systems trained on the largest corpora work on the smallest ones in some cases as well as systems trained on data directly from the smaller corpora themselves.

We identify a number of issues which make results achieved with different evaluation strategies and resources incomparable, or even incorrect. In our experimental design we consider the problems related to differences across corpora, the effects different cross-validation strategies have, and how parameter selection can be done. Our recommendation is to provide evaluations over different corpora, to use document-level cross-validation and to always select parameters on the training set.

We draw attention to the behavior of the F-score metric over corpora with differing pair distributions. The higher the relative frequency of interacting pairs is, the higher the performance can be expected to be. This is noticed both for the graph kernel method and for the naive co-occurrence baseline. Indeed, the strategy of just stating that all pairs interact leads to as high a result as 70% F-score on one of the corpora. We consider AUC as an alternative measure that does not exhibit such behavior, as it is invariant to the distribution of pairs. The AUC metric is much more stable across all the corpora, and never gives better results than random for approaches such as the naive co-occurrence.

Though we only consider binary interactions in this work, the graph representations have the property that they could be used to represent more complex structures than pairs. The availability of corpora that annotate complex interactions, such as the full BioInfer and GENIA, makes training a PPI extraction system for extracting complex interactions an important avenue of future research (see [38] for further discussion). However, how to avoid the combinatorial explosion following from considering triplets, quartets etc. remains an open question. Also, the performance of the current approaches may need to be yet improved before extending them to recognize complex interactions.

## Competing interests

The authors declare that they have no competing interests.

## Authors' contributions

AA designed the graph kernel, implemented it with the help of JB, and had the main responsibility for experiments. AA, FG, JB and SP explored suitable features and their representations. TP provided the sparse RLS algorithms and advice on kernel design. AA was the main author of the manuscript with contributions from all other authors, all of whom read and approved the final version.
